# Molecular Identification of Etiological Agents in Fungal and Bacterial Skin Infections: United States, 2020–2024

**DOI:** 10.3390/idr16060087

**Published:** 2024-11-18

**Authors:** Aditya K. Gupta, Tong Wang, Sara A. Lincoln, Hui-Chen Foreman, Wayne L. Bakotic

**Affiliations:** 1 Division of Dermatology, Department of Medicine, Temerty Faculty of Medicine, University of Toronto, Toronto, ON M5S 3H2, Canada; 2 Mediprobe Research Inc., London, ON N5X 2P1, Canada; twang@mediproberesearch.com; 3 Bako Diagnostics, Alpharetta, GA 30005, USA; slincoln@bakodx.com (S.A.L.); hforeman@bakodx.com (H.-C.F.); wbakotic@bakodx.com (W.L.B.)

**Keywords:** skin infection, molecular diagnostic, PCR, dermatophytosis, tinea, *Staphylococcus aureus*

## Abstract

**Background/Objectives**: Cutaneous infections of fungal and bacterial origins are common. An accurate diagnosis—especially concerning pathogens that are difficult to isolate on culture—can be achieved using molecular methods (PCR) with a short turnaround time. **Methods**: We reviewed records of skin specimens (superficial scrapings) submitted by dermatologists across the United States with a clinically suspected dermatitis. As per physician’s order, specimens were tested for infections either fungal (N = 4262) or bacterial (N = 1707) in origin. All unique specimens (one per patient) were subjected to real-time PCR assays where cases suspected of a fungal etiology were tested for dermatophytes, *Malassezia* and *Candida*, and cases suspected of a bacterial etiology were tested for *Streptococcus pyogenes*, *Staphylococcus aureus*, and the *mecA* gene potentially conferring β-lactam resistance. **Results**: Fungal agents were detected in 32.8% (SD: 4.5) of the submitted specimens, with most attributed to dermatophytes (19.3% (SD: 4.9)), followed by *Malassezia* (8.7% (SD: 2.8)) and *Candida* (2.9% (SD: 1.0)). Dermatophyte detection was more common in the elderly (≥65 years) compared to young adults (18–44 years) (OR: 1.8 (95% CI: 1.5, 2.2)), whereas *Malassezia* was more commonly detected in younger age groups (12.1–13.6%) than the elderly (5.6%). *Candida* was more frequently observed in females while dermatophytes and *Malassezia* were more frequently observed in males. Approximately one quarter of the submitted skin specimens tested positive for *S. aureus* (23.6% (SD: 3.4)), of which 34.4% (SD: 9.8) exhibited concurrent detection of the *mecA* gene. An *S. aureus* detection was more frequently observed in males (OR: 1.5 (95% CI: 1.2, 1.9)) and in children (OR: 1.7 (95% CI: 1.2, 2.5)). *Streptococcus pyogenes* was rarely detected. Among specimens positive for dermatophytes, 12.0% (20/166) showed co-detection of *S. aureus* and *mecA*, which is in contrast to 6.8% (70/1023) detected in samples without a fungal co-detection and 6.2% (8/130) in samples positive for *Malassezia*. **Conclusions**: PCR testing, when available, can be valuable as a part of routine care for diagnosing patients with clinically suspected skin infections. Further studies are warranted to survey the prevalence of resistant *S. aureus* isolates in dermatology outpatients, in particular with regard to the association with dermatophyte infections.

## 1. Introduction

Fungal and bacterial infections represent the majority of skin and subcutaneous diseases seen globally. Of the approximate 4.9 billion newly acquired cases in 2019, amounting to 43 million disability-adjusted life years lost, 1.6 billion cases were attributed to fungal infections and 1.1 billon cases were attributed to bacterial infections [1]. Fungal skin infections—predominately caused by dermatophytes—exhibit a higher disease burden in males and the elderly [1,2]. The age-standardized incidence of cutaneous bacterial infections—commonly caused by *Staphylococcus aureus*—have increased by 7.4% from 1990 to 2019 [3,4], and are associated with the highest mortality as compared to all other skin and subcutaneous diseases, particularly for females [1].

Mycology testing is recommended to confirm the diagnosis of a fungal skin infection (e.g., tinea corporis, tinea cruris) with culture being considered as the “gold standard”; however, this approach is complicated by long turnaround times of 2–4 weeks and a low sensitivity that can delay treatment [5]. Furthermore, the addition of antibiotics (e.g., chloramphenicol, gentamicin) in the culture medium—due to the slow growth of dermatophytes—may lead to the underdetection of concurrent bacterial colonization [6]. Direct microscopic examinations demonstrating fungal hyphae, despite its quick turnaround time and low costs, are not able to speciate the aetiological agent and may be used as first guidance while culture results are pending. To circumvent these limitations, newer molecular methodologies, including polymerase chain reaction (PCR), have become available, allowing the direct detection of genetic materials in clinical specimens [5]; a multiplexed design can simultaneously detect and identify multiple aetiological agents with a short turnaround time (1–2 days).

When bacterial skin and soft tissue infections are present, a purulent presentation may suggest a *S. aureus* infection; a non-purulent presentation may indicate a non-*S. aureus* infection (e.g., *Streptococcus pyogenes*) or non-infectious aetiologies [7]. A PCR diagnosis without requiring culture isolation can improve sensitivity, such as in the case of slow-growing colony variants [8]. In addition, the detection of molecular resistance markers by PCR—such as the *mecA* gene in *S. aureus* encoding a modified penicillin binding protein (PBP) conferring resistance to β-lactam antibiotics—assists in the identification of clinically significant strains that often elude culture diagnosis, and allows healthcare providers to make tailored treatment decisions [9].

Antibiotic resistance in *S. aureus* can be attributed to three distinct mechanisms: increased production of enzymes degrading penicillin-like antibiotics (β-lactamases), PBP mutations, and acquisition of PBP2a via horizontal gene transfer [10]. The latter is the predominant resistance mechanism found in clinical *S. aureus* isolates and can be used to characterize MRSA (methicillin-resistant *S. aureus*). PBPs exhibit transpeptidases activities and have essential functions in bacterial cell wall synthesis. PBP2a, encoded by the *mecA* gene that can be horizontally transferred by a mobile genetic element (SCC*mec*) with a low binding affinity to β-lactam antibiotics, serves as a rescue mechanism when intrinsic PBPs are inhibited [10]. Among the dermatology outpatient population, resistant *S. aureus* isolates reflective of community-acquired MRSA have been reported [11,12], which can manifest as a secondary infection in patients with atopic dermatitis, psoriasis, trauma, or diabetic foot. A recent meta-analysis by Elizalde-Jiménez et al. identified that ≥15% of *S. aureus* isolates from atopic dermatitis patients demonstrated reduced susceptibility to methicillin and oxacillin [13]; the *mecA* gene was also detectable in this patient population by sequencing and PCR [14,15]. In psoriasis patients, one study found that 21.9% (7/32) of *S. aureus* isolates cultivated from skin lesions were *mecA*-positive [16].

In the present study, we aim to detect and characterize skin infections of fungal and bacterial origins in the dermatology outpatient population through the use of multiplex real-time PCR (qPCR). Records of skin scrapings (2020–2024) submitted to a molecular diagnostic laboratory in the United States were reviewed with corresponding patient characteristics.

## 2. Materials and Methods

Superficial scrapings from patients suspected of infectious dermatitis were submitted by dermatologists across the United States to a CLIA-certified molecular diagnostic laboratory. Diagnostic results and patient demographic information (sex, age, clinic location) were reviewed spanning from January 2020 to May 2024 (3 years and 5 months). The present work constitutes a retrospective analysis of secondary data, which were de-identified. Molecular testing was provided as a part of routine, non-interventional, standard-of-care procedure, and, as such, does not represent a clinical trial requiring ethics overview and approval.

Following surface decontamination with an alcohol wipe to remove visible debris, superficial skin scrapings were collected via multiple unidirectional scrapes with a sterile scalpel blade or curette. The exfoliated material was captured in a Dermapak 2000 (DERMACO LTD) or placed in a sealed bag or other sterile container with a tightly fitting cap without fixative or medium, and transported at ambient temperature to the laboratory for processing. For DNA extraction, samples were placed in a beaded tube containing lysis buffer and homogenized on an Omni Bead Ruptor Bead-Mill prior to incubation and centrifugation. Then, DNA was extracted and purified on a Hamilton Microlab STAR workstation using a Mag-Bind Plant DNA DS kit (Omega Biotek) as per manufacturer’s instructions. The resulting eluate is used for PCR analysis. Samples were subjected to multiplex qPCR testing as per physician’s order; the superficial mycoses panel detects the presence of pan-dermatophytes, *Malassezia* and *Candida*, while the cutaneous infection panel detects the presence of *Streptococcus pyogenes*, *Staphylococcus aureus*, and the *mecA* gene.

Analysis was restricted to one specimen per patient. Quantitative variables were summarized using the mean and standard deviation (SD); analysis was conducted using one-way ANOVA with post-hoc Tukey adjustment where applicable, and two-tailed two-proportions Z test. Qualitative variables were summarized using counts and percentages; analysis was conducted using the chi-square test with Bonferroni correction. Odds ratios (ORs) with 95% confidence intervals (CIs) were calculated; 2-sided *p*-values were obtained as previously described by Altman and Bland [17]. Data curation and analysis were performed using Microsoft Excel (version 2301) and IBM SPSS Statistics (version 20). An alpha value of 0.05 was applied.

## 3. Results

A total of 6086 diagnostic records from 2020–2024 were reviewed, encompassing dry skin scrapings tested by multiplex qPCR. After de-duplication, there were 4262 unique specimens that were subjected to the fungal agent detection panel and 1707 unique specimens that were subjected to the bacterial agent and *mecA* gene detection panel. Cross-matching yielded 1404 unique specimens that were subjected to testing by both panels.

**Figure 1:**
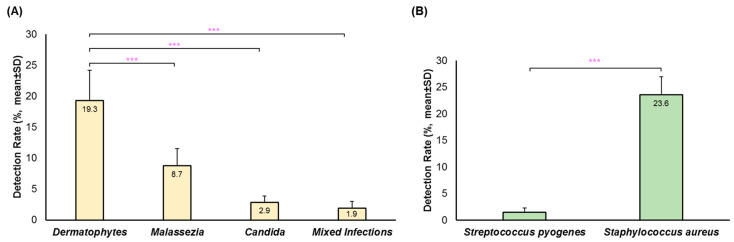
Detection rates for (**A**) fungal agents and (**B**) bacterial agents by multiplex qPCR. Results are stratified per year and presented as mean ± SD. *** *p* <0.001.

Suspected infectious dermatitis associated with fungal and bacterial agents were detected by multiplex qPCR (Figure 1). A fungal agent was identified in 32.8% (SD: 4.5) of the submitted specimens per year. The most common fungal agent detected was dermatophytes (19.3% (SD: 4.9)), which was significantly more common (*p* < 0.001) than *Malassezia* (8.7% (SD: 2.8)), *Candida* (2.9% (SD: 1.0)) and mixed fungal detections (1.9% (SD: 1.1)) (Figure 1A). In those cases where more than one organism was identified, the most common combinations were dermatophyte with *Malassezia* (57.0% (53/93)) and dermatophyte with *Candida* (25.8% (24/93)).

A significant association was found between patient sex (χ^2^ = 35.8, DF = 3, *p* < 0.001) and age (χ^2^ = 105.1, DF = 9, *p* < 0.001) with fungal agent identification results. Male patients exhibited a 40% higher likelihood for dermatophyte detections (OR: 1.4 (95% CI: 1.2, 1.6); 19.6% (382/1950) vs. 15.0% (343/2285)), and a 2-fold likelihood for *Malassezia* detections (OR: 2.1 (95% CI: 1.7, 2.6); 13.5% (263/1950) vs. 6.8% (156/2285)), than female patients (Table 1). Conversely, male patients were 40% less likely to be detected with *Candida* than female patients (OR: 0.6 (95% CI: 0.4, 0.9); 1.9% (38/1950) vs. 3.3% (76/2285)). An age-dependent increase was observed for dermatophyte detections and an age-dependent decrease was observed for *Malassezia* detections (Table 1). Compared to young adults (18–44 years), dermatophytes were 80% more likely to be detected in the elderly (≥65 years) (OR: 1.8 (95% CI: 1.5, 2.2); 22.8% (236/1035) vs. 14% (209/1496)). Similar results were observed for individuals aged 45–64 years compared to the 18–44-year age group (OR: 1.4 (95% CI: 1.1, 1.7)). *Malassezia* detections were the most common among children (<18 years) and young adults, detected at rates of 12.1% (61/505) and 13.6% (204/1496), respectively. Elderly patients exhibited a 60% lesser likelihood for *Malassezia* detections than young adults (OR: 0.4 (95% CI: 0.3, 0.5); 5.6% (58/1035) vs. 13.6% (204/1496)).

The bacterial agent most commonly detected was *S. aureus* (23.6% (SD: 3.4)) (Figure 1B); among these, the *mecA* gene was detected in an average of 34.4% (SD: 9.8) of samples reflecting a higher risk of β-lactam resistance. *Streptococcus pyogenes* was rarely detected (1.5% (SD: 0.8)). The *mecA* gene was present in a total of 28.4% (SD: 7.5) of skin specimens, of which 68.2% (SD: 4.4) were not found in association with *S. aureus*, possibly reflecting alternate *Staphylococcal* strains.

Overall, chi-square tests found no significant associations between patient characteristics and bacterial agent or *mecA* detection results. However, *S. aureus* detections exhibited a 50% statistically significant higher likelihood in male patients compared to female patients (OR: 1.5 (95% CI: 1.2, 1.9); 26.2% (204/780) vs. 19.3% (178/923)) (Table 2). *S. aureus* detections also exhibited a 70% higher likelihood in children (<18 years) than young adults (18–44 years) (OR: 1.7 (95% CI: 1.2, 2.5); 34.5% (58/168) vs. 23.3% (134/574)). Regional variations were observed for the detection of *mecA* as well as *mecA*-positive *S. aureus* (Table 2). Compared to the U.S. Midwest (40.9% (36/88)), *mecA* was less likely to be detected in the U.S. Northeast (OR: 0.4 (95% CI: 0.3, 0.6); 21.9% (137/625)) and West regions (OR: 0.4 (95% CI: 0.3, 0.7); 23.4% (61/261)). Similar results were observed for the co-detection of *mecA* and *S. aureus*.

**Figure 2:**
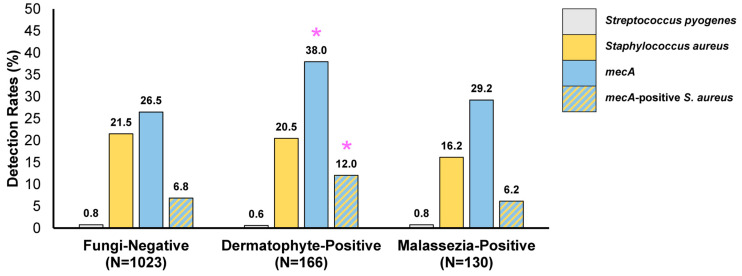
Detection of *Streptococcus pyogenes*, *S. aureus*, *mecA* and *mecA*-positive *S. aureus* in samples with or without concurrent detection of fungal agents. * *p* < 0.05 compared to the fungi-negative group.

A weak association was found between dermatophyte-positive samples and the detection of the *mecA* gene with and without *S. aureus* (Figure 2). The proportion of dermatophyte-positive samples detected with *mecA* (38.0% (63/166)) and *mecA* with *S. aureus* (12.0% (20/166)) were significantly higher (*p* < 0.05) than that observed in fungi-negative samples (26.5% (2711/1023) and 6.8% (70/1023), respectively). In contrast, skin specimens positive for *Malassezia* did not exhibit significant differences in *mecA* (29.2% (38/130)) and *mecA* with *S. aureus* (6.2% (8/130)) compared to specimens without the detection of fungal agents.

## 4. Discussion

Dermatophytes (*Trichophyton*), *Streptococcus pyogenes*, and *S. aureus* are associated with skin infections. Through the use of an efficient molecular methodology for diagnosis, we provide an updated perspective on the detection of the aforementioned etiological agents in the United States among the dermatology outpatient population.

Consistent with our current understanding, dermatophytes are the predominant cause of fungal skin infections; in our cohort, we found dermatophyte detections to be more common in male patients and the elderly. The higher prevalence of dermatophytes in the elderly could be explained by an elevated co-morbidity burden, including immunosuppression, diabetes, and obesity [5]. Male and elderly patients are at a higher risk for developing tinea pedis; in one recent study, tinea pedis was diagnosed in 16.7% of outpatients aged ≥70 years and in 19.4% of male patients [18]. Male patients also exhibit higher risk for developing tinea cruris [19]; the management of this condition can be complicated by the development of a secondary bacterial infection or skin maceration causing pain. A new dermatophyte strain, identified as *T. mentagrophytes* ITS genotype VII, has been reported as an agent of sexual transmission in men who have sex with men [20,21].

*Malassezia* is not commonly reported as a cause for skin infections. Obtaining an isolate is difficult owing to the requirement of specialized media containing lipids [22]. In pityriasis (tinea) versicolor, the pathogenic role of *Malassezia* is evidenced by the mycelial growth on direct microscopic examination that can invade the stratum corneum, whereas in other dermatologic conditions (e.g., atopic dermatitis, seborrheic dermatitis), *Malassezia* may be detected as a colonizer that triggers inflammation [23]. Our findings show an average of 8.7% of outpatients were positive for *Malassezia*, which were more common among younger age groups. This differential detection rate can be explained by the lipolytic activities of *Malassezia* that disproportionately target younger individuals with higher levels of sebum production and hyperhidrosis [24]. Possible cutaneous candidiasis was also detected at a rate 2.9%, which affected females more frequently than males (3.3% vs. 1.9%) with a 40% higher likelihood. The elevated risk in females may be linked to higher levels of estrogen altering the metabolic profile in *C. albicans* associated with the transition into its hyphal form [25], as well as impairing the host innate immune response [26].

Bacterial identification was predominately *S. aureus*, detected in an average of 23.6% of the submitted specimens. This is consistent with our current understanding of *S. aureus* as the main etiological agent in bullous and non-bullous impetigo in the Northern Hemisphere as opposed to *Streptococcus pyogenes* [27,28]. *S. aureus* was more commonly detected in children (34.5%) compared to older age groups (19.6–23.3%); this difference may reflect the higher propensity for children in developing impetigo [29]. Other plausible clinical diagnoses for primary *S. aureus* infections include abscess and folliculitis [3]. A disrupted skin microflora in atopic dermatitis patients also increases the risk of *S. aureus* infections, which in turn exacerbates inflammation [30].

In this study, we observed an elevated propensity for detecting the *mecA* gene in association with *S. aureus* when the skin specimen is positive for dermatophytes. Due to a limited sample size and lack of clinicopathological correlation, we cannot ascertain the significance of this finding as *S. aureus* naturally inhabits the mucous membranes of the nasal passage and may transiently colonize the skin without symptoms [31]. *S. aureus*, including the methicillin-resistant phenotype, has been reported to colonize skin lesions in dermatology outpatients, including those with or without a skin and soft tissue infection [11,32]. In one U.S. study, 36% of *S. aureus* isolates obtained from one dermatology outpatient clinic were characterized as methicillin-resistant *S. aureus* (MRSA) [11]. In a case report, an infant with tinea capitis presenting with a purulent scalp infection was detected with community acquired MRSA [33]; however, differentiation between a secondary MRSA infection and mere colonization was not possible. Although no dermatophyte isolates grew on culture initially, authors utilized PCR testing that led to the identification of *T. verrucosum* [33].

A dermatophyte infection, in particular for cases of chronic or severe infections in high-risk individuals (e.g., immunocompromised, diabetics), can lead to a secondary bacterial infection that may warrant the use of oral and topical antibiotics [5]. A pre-existing skin lesion due to the keratinolytic and lipolytic activities of dermatophytes and modulation of the host immune response may create a point of entry leading to a secondary *S. aureus* infection [3,34]. Furthermore, clinical dermatophyte isolates (*T. rubrum, T. mentagrophytes*) have demonstrated antibiotic-producing potential in vitro, thereby increasing the potential to induce penicillin-resistance in *S. aureus* [35,36]. A recent study by Larsen et al. isolated *T. erinacei* from European hedgehogs, their results demonstrate the production of two β-lactam antibiotics associated with the natural selection of MRSA [37]. In view of the above, it is conceivable that patients with chronic, severe dermatophytoses may develop a secondary *S. aureus* infection including the possibility of MRSA. Further studies are warranted to confirm this observation and its significance.

The present study is limited by the retrospective design and lack of additional clinical correlations such as patient symptoms and the location of the skin swab. We could not exclude the possibility of sampling bias; for instance, the higher likelihood of detecting *Candida* in females may be due to a higher degree of clinical suspicion in case of vulvovaginitis, whereas males may be less likely to be swabbed for *Candida* in comparison. Since only skin scrapings were collected without biopsies, our findings are less relevant in case of subcutaneous infections. Due to the convenience sampling approach, this cohort of patients may overrepresent those with high disease severity than the background population. Further prospectively designed studies with a well-defined patient population and a standardized sampling approach are warranted to confirm these findings.

## 5. Conclusions

In contrast to the use of traditional fungal or bacterial cultures for the diagnosis of skin infections, the use of PCR testing can significantly improve diagnostic sensitivity while shortening the turnaround time. Direct microscopy remains a quick and cost-effective method; however, this method alone only raises clinical suspicion while a definitive diagnosis would still require culture or molecular diagnostics such as PCR. Through a retrospective analysis of dermatology outpatients who presented with clinically ambiguous dermatitides and whose skin specimens were subjected to multiplex qPCR testing, including 4262 skin specimens tested for fungal agents and 1707 skin specimens tested for bacterial agents, our observations reaffirm existing knowledge on the primary etiological agents and patient risk factors. The high prevalence of the *mecA* gene in *S. aureus* (34.4%) detected in skin specimens highlights the need for further research into resistance development and its impacts for clinical practice. The potential causal relationship between a dermatophyte infection and a secondary *S. aureus* infection—including MRSA—should be investigated in future studies.

## Figures and Tables

**Table 1:** Fungal agent detection results stratified per patient characteristics.

Parameter	Dermatophyte			*Malassezia*			*Candida*			Mixed Detection			
	N	%	OR (95% CI)	N	%	OR (95% CI)	N	%	OR (95% CI)	N	%	OR (95% CI)	
**Sex**													
Male	382	19.6	**1.4 (1.2, 1.6)**	263	13.5	**2.1 (1.7, 2.6)**	38	1.9	**0.6 (0.4, 0.9)**	58	3.0	**2.0 (1.3, 3.1)**	
Female	343	15.0	Referent	156	6.8	Referent	76	3.3	Referent	34	1.5	Referent	
**Age Group**													
<18	57	11.3	0.8 (0.6, 1.1)	61	12.1	0.9 (0.6, 1.2)	8	1.6	0.7 (0.3, 1.5)	4	0.8	0.5 (0.2, 1.5)	
18–44	209	14.0	Referent	204	13.6	Referent	35	2.3	Referent	23	1.5	Referent	
45–64	225	18.4	**1.4 (1.1, 1.7)**	99	8.1	**0.6 (0.4, 0.7)**	35	2.9	1.2 (0.8, 2.0)	22	1.8	1.2 (0.6, 2.1)	
≥65	236	22.8	**1.8 (1.5, 2.2)**	58	5.6	**0.4 (0.3, 0.5)**	36	3.5	1.5 (0.9, 2.4)	44	4.3	**2.8 (1.7, 4.7)**	
**Region**													
Northeast	237	14.9	**0.7 (0.5, 0.9)**	150	9.5	1.1 (0.8, 1.7)	40	2.5	0.8 (0.4, 1.6)	26	1.6	0.5 (0.3, 1.1)	
Midwest	76	21.1	Referent	30	8.3	Referent	11	3.1	Referent	11	3.1	Referent	
South	318	17.1	0.8 (0.6, 1.0)	204	10.9	1.4 (0.9, 2.0)	49	2.6	0.9 (0.4, 1.7)	45	2.4	0.8 (0.4, 1.5)	
West	93	23.6	1.2 (0.8, 1.6)	34	8.6	1.0 (0.6, 1.7)	11	2.8	0.9 (0.4, 2.1)	9	2.3	0.7 (0.3, 1.8)	

ORs with a statistically significant 95% CI (*p* < 0.05) are bolded.

**Table 2:** Detection of *S. aureus*, *mecA*, and *S. aureus* with concurrent *mecA* stratified per patient characteristics.

Parameter	*S. aureus*			*mecA*			Co-Detection *S. aureus* and *mecA*		
	N	%	OR (95% CI)	N	%	OR (95% CI)	N	%	OR (95% CI)
**Sex**									
Male	204	26.2	**1.5 (1.2, 1.9)**	253	32.4	1.1 (0.9, 1.4)	76	9.7	1.3 (0.9, 1.8)
Female	178	19.3	Referent	272	29.5	Referent	70	7.6	Referent
**Age Group**									
<18	58	34.5	**1.7 (1.2, 2.5)**	44	26.2	0.8 (0.6, 1.2)	14	8.3	0.9 (0.5, 1.7)
18–44	134	23.3	Referent	172	30.0	Referent	51	8.9	Referent
45–64	98	20.0	0.8 (0.6, 1.1)	149	30.3	1.0 (0.8, 1.3)	42	8.6	1.0 (0.6, 1.5)
≥65	93	19.6	0.8 (0.6, 1.1)	161	34.0	1.2 (0.9, 1.6)	40	8.4	0.9 (0.6, 1.5)
**Region**									
Northeast	113	18.1	0.6 (0.4, 1.0)	137	21.9	**0.4 (0.3, 0.6)**	29	4.6	**0.3 (0.2, 0.7)**
Midwest	23	26.1	Referent	36	40.9	Referent	11	12.5	Referent
South	180	25.2	1.0 (0.6, 1.6)	288	40.4	1.0 (0.6, 1.5)	86	12.1	1.0 (0.5, 1.9)
West	61	23.4	0.9 (0.5, 1.5)	61	23.4	**0.4 (0.3, 0.7)**	19	7.3	0.5 (0.3, 1.2)

ORs with a statistically significant 95% CI (*p* < 0.05) are bolded.

## Data Availability

Restrictions apply to the availability of these data. Data were obtained from Bako Diagnostics (Alpharetta, GA, USA) and are available from A.K.G with the permission of Bako Diagnostics.
